# Mesoporous Silicon Particles Favor the Induction of Long-Lived Humoral Responses in Mice to a Peptide-Based Vaccine

**DOI:** 10.3390/ma11071083

**Published:** 2018-06-26

**Authors:** Gabriela Navarro-Tovar, Denisse Rocha-García, Alejandra Wong-Arce, Gabriela Palestino, Sergio Rosales-Mendoza

**Affiliations:** 1 Laboratorio de Biofarmacéuticos Recombinantes, Facultad de Ciencias Químicas, Universidad Autónoma de San Luis Potosí, Av. Dr. Manuel Nava 6, San Luis Potosí 78210, Mexico; gnavarro@conacyt.mx (G.N.-T.); awa_2892@hotmail.com (A.W.-A.); 2 Sección de Biotecnología, Centro de Investigación en Ciencias de la Salud y Biomedicina, Universidad Autónoma de San Luis Potosí, Av. Sierra Leona 550, Lomas 2ª. Sección, San Luis Potosí 78210, Mexico; qsrm@hotmail.com (D.R.-G.); palestinogabriela@uaslp.mx (G.P.); 3 Consejo Nacional de Ciencia y Tecnología, CONACyT, Av. Insurgentes Sur 1582, Col. Crédito Constructor, Del. Benito Juarez, Ciudad de Mexico 03940, Mexico; 4 Laboratorio de Biopolímeros y Nanoestructuras, Facultad de Ciencias Químicas, Universidad Autónoma de San Luis Potosi, Av. Dr. Manuel Nava 6, San Luis Potosí 78210, Mexico

**Keywords:** humoral response, receptor for advanced glycation end products, adjuvant, peptide vaccine, vaccine delivery vehicle

## Abstract

Vaccinology faces the challenge of developing improved immunization approaches that are able to induce long-term immunity with the desired Th profile according to the pathology. In this context, new vehicles for efficient antigen delivery that exert adjuvant effects play a critical role in addressing this goal. Herein, mesoporous silicon particles (PSiP) were assessed as carriers for a peptide-based vaccine targeting the receptor for advanced glycation end products (RAGE), which is a relevant receptor in Alzheimer´s disease and other diseases. A RAGE peptide was adsorbed onto PSiP (PSiP vaccine) and administered to BALB/c mice, leading to immune responses that were similar in magnitude to those induced by the soluble peptide. However, the response induced by PSiP lasted for a significantly longer period when compared with the behavior of the group immunized with the peptide alone. Therefore, PSiP are proposed as carriers to enhance immune memory, which is critical in vaccination. This study opens interesting perspectives related to the application of PSiP in vaccinology.

## 1. Introduction

Although subunit vaccines offer high safety and specificity, a frequent challenge for such type of vaccines is the poor immunogenic activity that demands the use of adjuvants to enhance the induced immune response [1]. Several types of nanomaterials have been explored as delivery systems with adjuvant activity such as multimeric proteins [2], organic nanoparticles [3], and metallic nanoparticles [4].

Over the last few decades, interest in nanovaccines has increased, and promising results have been found. The success of implementing micromaterials or nanomateriasl in biomedical technologies relies on their biocompatibility and physicochemical properties. In this sense, mesoporous silicon micromaterials and nanoparticles have the following interesting characteristics: (i) a high surface area/volume ratio with Si-H_x_ and Si-O_x_ chemical bonds on the surface that facilitate biomolecules loading [5]; (ii) a small-size tunable porous structure (less than 50 nm in diameter) where biomolecules can be loaded, allowing a fine controlled delivery in the biological system [6]; (iii) biocompatibility; and (iv) biodegradable with silicic acid (Si(OH)_4_) as the final degradation product, which is an inorganic component of bones [7]. Sailor’s research group reported that mesoporous silicon particles (PsiP) are completely degraded in 24 h under in vitro physiological conditions (pH = 7.4 and 37 °C), and the silicon accumulated after intravenous injection of 20 mg/kg*_mouse_* was cleared from the body within a period of one to four weeks [8,9].

Several studies have assessed PSiP as a potential vehicle of conventional drugs [10,11] and biopharmaceuticals [8,12], and as an imaging diagnostic agent [13,14,15], and pointed out such materials as attractive candidates. In the vaccinology arena, it has been shown that PSiP can be functionalized with relevant ligands to efficiently enhance the phagocytosis of microparticles by dendritic cells, inflammasome activation, the upregulation of co-stimulatory and major histocompatibility complex (MHC) molecules, migration to lymphatic tissue, and cellular interactions that lead to T-cell activation [16,17]. Jiménez-Periáñez et al. [17] demonstrated that PSiP enhanced the MHC class I presentation of viral-specific CD8^+^ T cell epitopes by human monocyte-derived dendritic cells (MDDCs) to CD8^+^ T lymphocytes. Moreover, it has been reported that porous silicon nano/microparticles loaded with an antigen of the human epidermal growth factor receptor 2 (HER2, a member of the HER family of proteins and overexpressed in some cancer), greatly enhance cross-presentation in DCs ex vivo and activate type I interferon (IFN-1) response with the subsequent induction, following DCs transfer, of a potent CD8 T cell-dependent anti-tumor immunity in mice [18].

The receptor for advanced glycation end products (RAGE) is a relevant target for the development of immunotherapies. RAGE consists of three main regions: the extracellular domain (amino acid residues 23–342) that interacts with the ligands, a transmembrane domain (residues 343–363), and a short intracellular domain (residues 364–404) involved in transmembrane signaling. The extracellular region is composed of three immunoglobulin-like domains: a V-type domain, and two C-type domains (C1 and C2) [19]. It has been reported that a vaccine targeting the V domain induced humoral responses that are able to block the activity of RAGE, which is associated with the progression of inflammatory disorders, tumor outgrowth, diabetic complications, cardiovascular diseases, and Alzheimer’s disease [20].

Although PSiP are described as a potential adjuvant in immunotherapy and vaccines, this concept has not been assessed in schemes comprising direct immunization of test animals, which is the most practical approach in vaccinology. Herein, we evaluated whether PSiP act as an adjuvant for a vaccine targeting RAGE. A peptide-based vaccine targeting RAGE was developed by the adsorption of a RAGE peptide (RAGEp) from the extracellular region of RAGE onto PSiP. The humoral response induced in test mice by the RAGEp/PSiP vaccine was analyzed and compared with that induced by the RAGE peptide alone.

## 2. Results

### 2.1. Characterization of PSiP Particles

**Figure 1:**
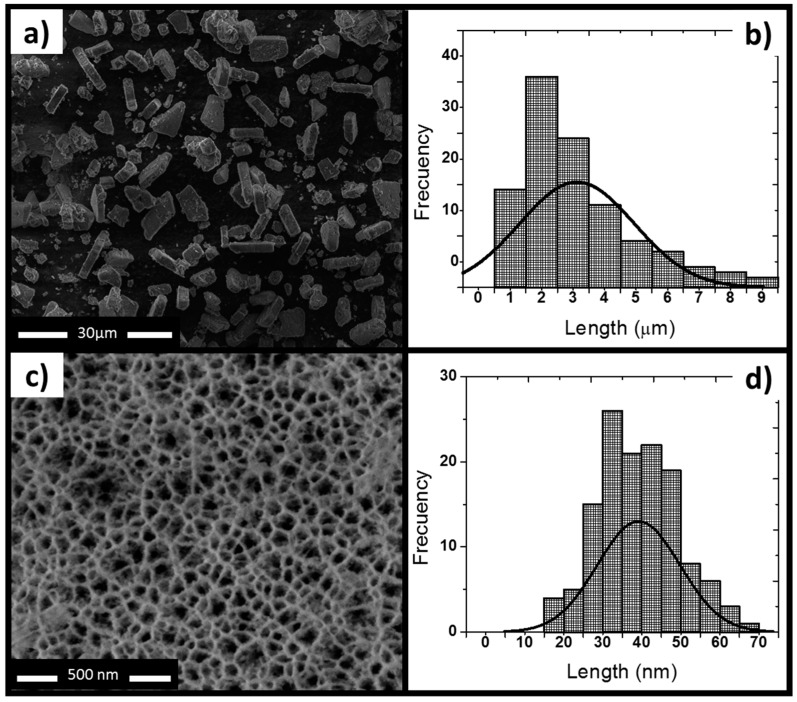
Morphology characterization of mesoporous silicon particles (PSiP). (**a**) SEM top view of PSiP showing irregular discoid structures; (**b**) Size distribution of PSiP. The statistical analysis of the SEM image displays an average particle size of 3 ± 1 μm; (**c**) Front view of a porous layer; (**d**) Pore size distribution of PSiP. The statistical analysis of the SEM image indicates an average pore size of 40 μm.

**Figure 2:**
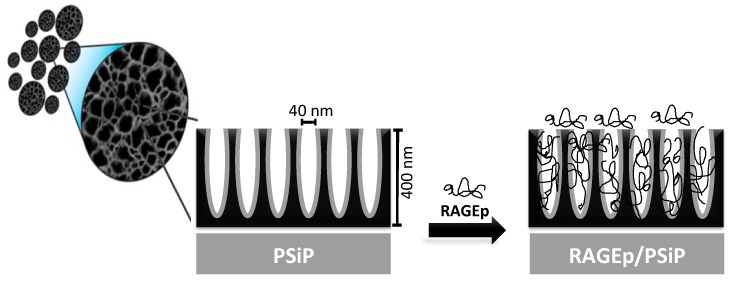
Schematic representation of the adsorption of RAGEp onto the PSiP surface and pores in the particle structure. The PSiP are porous irregular discoids with a size distribution of 3 ± 1 µm, a thickness of ~400 nm, and a pore size of 40 nm. The anionic PSiP surface interacts with the cationic RAGEp molecules through electrostatic and/or ion dipole forces.

This study reports the characterization of a single synthetized batch of thermally oxidized PSiP. The SEM images showed that the oxidized PSiP had irregular flat discoid shape (Figure 1a), and the statistical analysis of a SEM micrography of 150 particles determined a size distribution of 3 ± 1 µm (Figure 1b) and a thickness of ~400 nm. The sonication cycles, including the time and number of cycles, are the main factors in the resulting shape and particle size [21,22]. Moreover, the SEM analysis demonstrated that the PSiP surface presented homogeneous, cylindrical porous structures (Figure 1c) with an average pore size of 40 nm (Figure 1d); this confirms the presence of a mesoporous material. Figure 2 represents the porous material obtained by electrochemical etching and subsequent sonication, as well as the oxidized PSiP material. The thermal oxidation had a minimal effect on the pore size; however, it was conducted in order to stabilize and provide a negative charge to the surface of the PSiP, as described by several authors [22,23,24].

**Figure 3:**
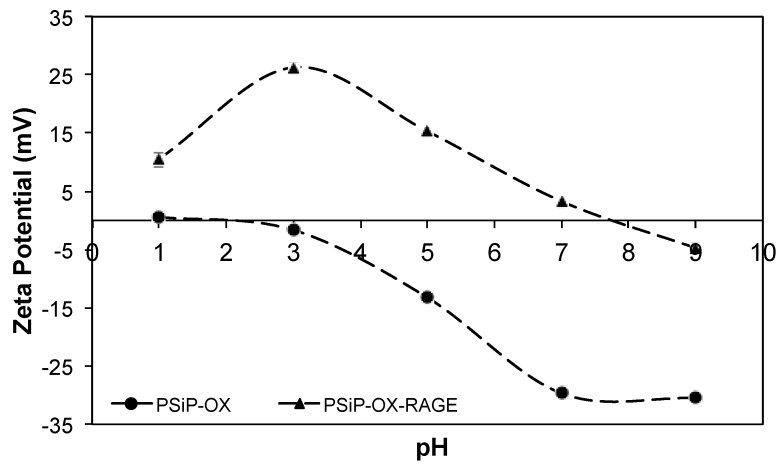
Titration curve for PSiP (•) and PSiP-RAGEp (▲). Titrations were performed in deionized water at different pH values. The selected titrants were HCl and NaOH.

Moreover, the PSiP surface charge was measured at different pH conditions demonstrating a negatively charged surface. In the pH range from 7 to 9, the PSiP showed a surface charge of −33 mV, which is the expected charge under physiological conditions (Figure 3). Additionally, no aggregation of the PSiP was found in the established experimental conditions. According to the DLVO theory (named after Boris Dejaguin, Lev Landau, Evert Verwey and Theodor Overbeek) in which is described the influence of Van der Waal and Coulombic forces in the colloidal or particle stability, a zeta potential value of ±30 mV is found for stable particles [25]. In this study, the oxidized PSiP are stable in a pH range from 7 to 9. On the other hand, a chemical characterization of the PSiP surface was carried out using Fourier transform infrared (FT-IR) analysis. The resulting FT-IR spectrum for oxidized PSiP (a) showed a medium and broad band at 3370 cm^−1^ due to O–H stretching from the Si–O–H bond. Furthermore, the IR spectrum revealed a strong and broad band at 1066 cm^−1^ for the asymmetric stretching of the Si–O–Si surface bond, a weak band at 2150 cm^−1^ that corresponds to the stretching signal of OSiH_3_, a broad and strong band at 1066 cm^−1^ with a slight shoulder at 1215 cm^−1^ due to stretching of the Si–O–Si bond, and two weak signals at 767 cm^−1^ and 657 cm^−1^ corresponding to deformational vibration of O_n_Si-H_x_ [26,27,28].

### 2.2. Evaluation of RAGEp Adsorption onto the PSIP Surface

In this study, RAGEp was selected as the vaccine target implicated in therapies against several pathologies. A basic study on the interaction of RAGEp and PSiP was performed before the biological evaluation of the resulting RAGEp/PSiP conjugate.

**Figure 4:**
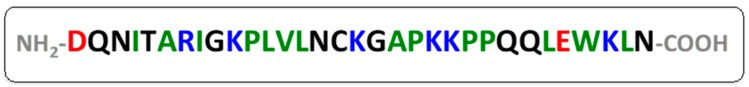
Sequence of RAGEp. The receptor for advanced glycation end products peptide (RAGEp) is a synthetic peptide comprising the amino acids 23–54 from the human RAGE, at the extracellular region. Red indicates COOH residues with negative charge (COO^−^) at a physiological pH, and blue stands for NH_2_ residues with positive charge (NH_3_^+^) at a physiological pH. Green specifies hydrophobic uncharged residues, and black represents other residues.

**Figure 5:**
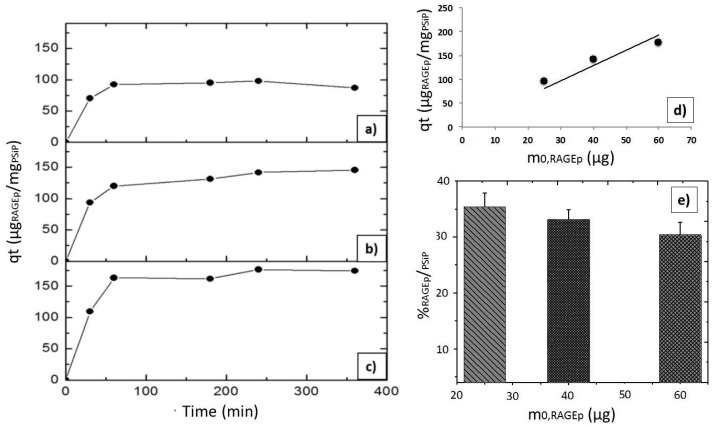
RAGEp adsorption onto PSiP. Adsorption experiments were carried out using 100 μg of PSiP and different initial RAGEp amounts in μg: (**a**) 25, (**b**) 40, and (**c**) 60. A final volume of 250 μL was attained with PBS solution (pH = 7.4). All of the suspensions were stirred at 6 °C during the experiment. The concentration of soluble RAGEp was determined by measuring absorbance at λ_max_ = 199 nm. All of the experiments were conducted by triplicate, and the %SD were as follows: (**a**) 1.7–3.0%, (**b**) 0.1–2.5%, and (**c**) 2.0–3.0. (**d**) Linear concentration-dependent RAGEp adsorption. Data obtained after 4 h of experiment in the described conditions, r^2^ = 0.78. (**e**) Percentage of RAGEp adsorbed onto the PSiP surface (%_RAGEp/PSiP_) with respect to the initial RAGEp amount (m_0,RAGEp_). Data are the average values of three experiments.

**Figure 6:**
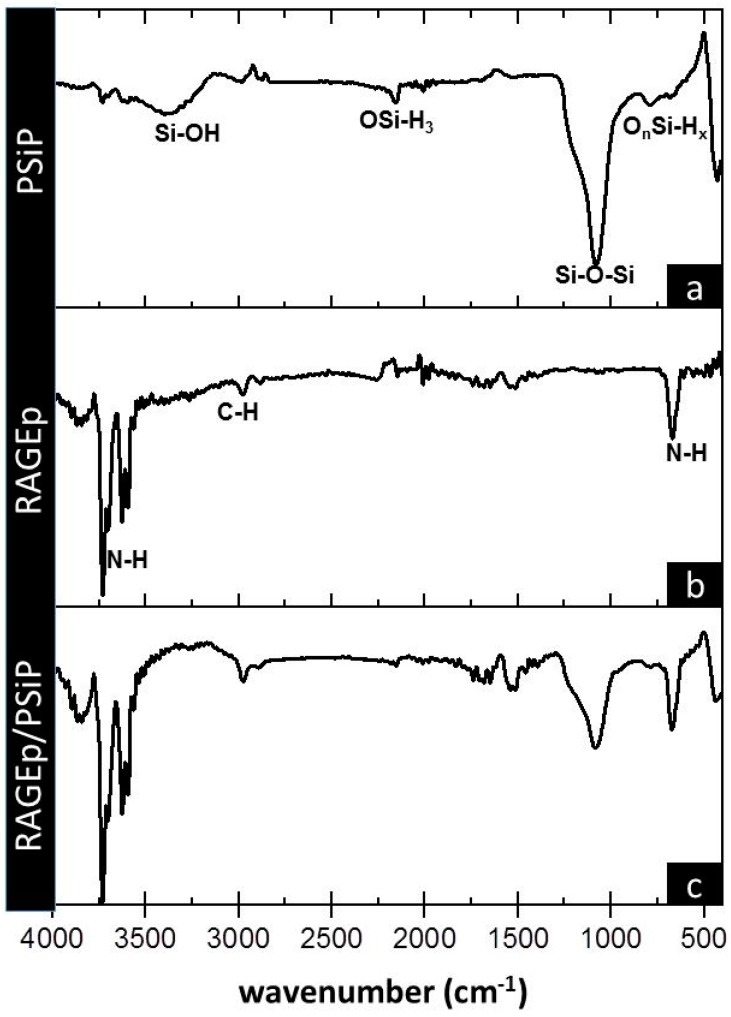
Infrared spectra of (**a**) PSiP, (**b**) RAGEp, and (**c**) RAGEp/PSiP conjugates. IR analyses were carried out after four hours of the adsorption experiment at the described conditions. PSiP and RAGE–PSiP were centrifuged, and pellets were washed twice with phosphate-buffered saline solution (PBS) before Fourier transform infrared (FT-IR) analyses. Chemical groups are assigned below relevant bands.

According to the characteristics of both PSiP and RAGEp (Figure 4), it is expected that the peptide interacts with the negatively charged PSiP surface (Figure 2). Figure 5a–c shows the amount of RAGEp adsorbed onto the PSiP surface (*qt*) in terms of μg_RAGEp_/mg_PSiP_ versus time. For all of the conducted experiments, the average μg_RAGEp_/mg_PSiP_ values significantly increased within the first two hours of study, reaching a plateau at further time points. Linear concentration dependent adsorption was observed in the range of 25–60 µg (Figure 5d), whereas saturation was observed at peptide amounts above 60 µg (data not shown). The analyses at 4 h showed no variations in *qt* with data obtained at 24 h (data not shown). The %_RAGEp/PSiP_ values calculated at 4 h are shown in Figure 6e. The low molecular weight of RAGEp (3.6 kDa, which represents an approximate size of 2 nm) ensures that the peptide is able to enter the cavities of PSIP while its positive charge ensures an electrostatic interaction with the negatively charged PSiP surface. Thus, both the chemistry of the oxidized PSiP surface and the cavities in these silicon particles facilitated peptide loading [29,30,31]. The physiological pH of 7.4 favors a negative charge onto the PSiP surface, while RAGEp possesses a positive charge at the same pH (*pI* = 10.62) due to the addition of protons in the –NH_2_ residues (Figure 2). Figure 4 evidences a drastic change in the zeta potential of RAGEp/PSiP (+2 mV) with respect to PSiP alone (−33 mV), suggesting electrostatic or ion–dipole interactions between the peptide and particle surface [30,31,32].

The peptide–particle surface interaction is also observed in the RAGEp/PSiP IR spectrum (Figure 6c), with a decrease in the intensity of several bands with respect to PSiP alone (Figure 6a) and the incorporation of signals corresponding to RAGEp. The RAGEp alone spectrum is shown in Figure 6b with characteristic asymmetric and asymmetric stretching bands at 3698 cm^−1^, 3592 cm^−1^, and 667 cm^−1^ corresponding to NH_2_, and a stretching band at 2989 cm^−1^ attributed to C–H. The possible intermolecular forces involved in the RAGEp adsorption are listed in Table 1, where electrostatic and/or ion dipole forces are related with changes on the IR signals.

### 2.3. The RAGEp/PSIP Vaccine Induces Long-Lasting Humoral Response in BALB/c Mice

**Figure 7:**
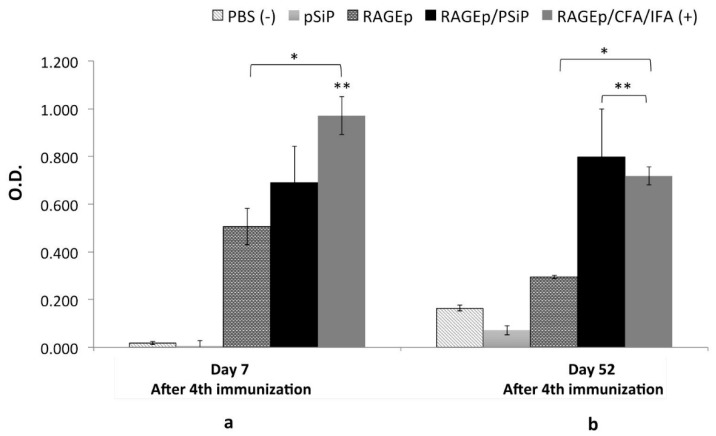
Long-lasting immune responses in BALB/c mice immunized with RAGEp/PSiP. Mice received four subcutaneous doses of one of the following treatments: PBS as negative control, RAGEp alone, PSiP, RAGEp/PSiP, or RAGEp/complete Freund’s adjuvant (CFA)/incomplete Freund’s adjuvant (IFA) as positive control. Serum samples were obtained at day 0 (before the first immunization) and at days 7 (**a**) and 52 (**b**) after the fourth immunization. Antibody levels were determined by ELISA, and data are presented as the average optical density (O.D.) (1:20 dilution) at either day 7 or 52 min average O.D. at day 0. Statistical differences (*p* < 0.05) versus the group treated with PBS are indicated by an asterisk (*), while statistical differences versus the group treated with RAGEp alone are indicated by double asterisk (**).

The immune response upon immunization with the RAGEp/PSiP vaccine was assessed in test mice under a scheme comprising four weekly subcutaneous immunizations. Groups immunized with PBS, RAGEp alone, or RAGEp along with complete Freund’s adjuvant–incomplete Freund’s adjuvant (CFA/IFA) were used as controls. Immunization with RAGEp alone induced significant humoral responses, whose magnitudes on day 7 after the last boost (*p* < 0.05) were statistically undistinguishable from the response of the groups treated with the RAGEp/PSiP vaccine. Besides, the humoral response induced by the RAGEp/CFA/IFA (positive control) vaccine was significantly higher than the immune response produced by the RAGEp/PSiP vaccine (Figure 7a). Interestingly, when humoral responses were measured 52 days after receiving the last boost, the RAGEp group showed a significant decrease (about two times) compared with the previous anti-RAGE antibody levels, whereas the RAGEp/PSiP-treated group showed sustained antibody levels, whose magnitude was statistically undistinguishable from the response attained in the RAGEp/CFA/IFA adjuvanted group (Figure 7b). Particularly, the response of the RAGEp/PSiP group was statistically higher (about three times) than the response achieved by the RAGEp group at 52 days, suggesting the induction of long-lived humoral responses.

## 3. Discussion

In the present study, we explored the ability of PSiP to serve as adjuvant/carrier for a peptide vaccine whose target antigen consists of a segment of the extracellular domain of RAGE. It was aimed at the induction of RAGE-blocking antibodies in the host looking to achieve therapeutic effects in several conditions, including cancer and Alzheimer’s disease. For some therapeutic applications of protein–nanoparticle conjugates, the use of proteins can be replaced by the synthetic peptides that achieves the same or a more specific biological effect. The small size of peptides reduces allow to increase the number of peptide molecules per nanoparticle [34]. Furthermore, the use of PSiP as carrier protects the peptide from dilution and degradation and favors antigen presentation and lymphocyte activation processes [30].

The electrochemical etching synthesis method is extensively reported in the literature to obtain microparticles and nanoparticles with a range of size from 0.01 µm to 20 µm [35,36]. The production of PSiP follows a “*top–down*” method, where the particles are obtained by mechanical, sonication, or ultrasonication methods. Therefore, the particles end up with irregular discoid shape. In this work, the particle size is similar to the values reported by Jiménez-Periáñez et al. [17] (500 nm to 5 µm) and Xia et al. [18] (400 nm thickness and 1 µm). Both reports suggest the potential of porous silicon microparticles as immunogenic carriers in in vitro and ex vivo studies.

The adsorption of biomolecules, such as peptides, is driven by the physicochemical properties of both particles and biomolecules. Thus, the particle size, pore size, pore density in the material, and the chemical groups onto the surface are all parameters of the particles that determine the adsorption profiles [30,37]. It has been demonstrated that both pore size and pore density influence the protein adsorption [38]. Moreover, it has been reported that the particle size strongly influences thermodynamic (e.g., molar Gibbs free energy adsorption) and kinetic parameters (e.g., adsorption constant rate) during the adsorption of molecules onto metallic oxide nanoparticles [39]. On the other hand, the polarity of the peptide, isoelectric point, size, and concentration in solution play an important role in the biomolecule’s interaction with the particle surface. Additionally, the solvent, salts, and pH could affect this interaction.

In the present study, we first assessed the RAGEp loading degree of the PSiP under the same conditions that were used to prepare the RAGEp/PSiP vaccine. Our results show the adsorption of RAGEp onto the PSiP surface possibly through electrostatic forces or ion–dipole forces. Higher %_RAGEp/PSiP_ values (30–38%) were achieved when the RAGEp initial amount was ≤60 μg. Both surface interaction and distribution in the PSiP pores could promote the loading (Figure 5). Kaasalainen et al. [32] suggested that small peptides with isoelectric point (*pI)* values higher than 8 and bearing positively charged amino acids (lysine (K) and arginine (R)) interact with silicon-derived particles such as silica particles through electrostatic forces with the Si–O and O–Si–O groups on the silica particle surface. Moreover, the RAGEp adsorption appears to be dependent on the initial amount of peptide (Figure 7). In this sense, the cationic RAGEp could be ion pairing with the negatively charged O_n_Si–H_x_, Si–O, and O–Si–O groups on the PSiP surface.

The loading of biomolecules into PSiP is usually achieved by simply mixing both components in water or physiological solutions, leading to an interaction mediated by intermolecular forces. In biological evaluations, as the present study reports, the control of key parameters such as temperature, pH, concentration, agitation, and time of contact is relevant to obtain a particulated formulation with reproducible characteristics (loading degree and adsorption profile) [40,41].

This basic RAGEp adsorption study was relevant to suggest the physical peptide–PSiP interactions and define the optimal experimental conditions for the peptide-based vaccine preparation, in which the minimal amount of RAGEp could be added to PSiP in suspension to achieve the maximum %_RAGEp/PSiP_. The results also determined the suitable dose of soluble RAGEp to be used in mice immunization schemes.

We next explored the immunogenic activity of the RAGEp/PSiP vaccine in test mice. Interestingly, the use of PSiP as vaccine carriers led to long-lived humoral responses, as evidenced by the significantly higher antibody levels observed on day 52 after the last boost in comparison to those attained with the group treated with RAGEp alone. This effect could be associated with the previously reported immunostimulatory effects of PSiP such as the enhancement of phagocytosis by DCs, the upregulation of co-stimulatory and MHC molecules, and migration to lymphatic tissue; as well as cellular interactions leading to T cell activation [16,17]. We hypothesize that PSiP favored the expansion of Th1 cells; thus, further studies will aim at determining if the use of PSiP in this peptide-based vaccine modifies the expansion of specific Th populations.

Since immunotherapies are becoming an important trend to treat non-communicable diseases, the RAGEp/PSiP vaccine will serve as a useful model in the development of chronic conditions such as Alzheimer’s disease and cancer. For instance, this vaccine is a promising candidate for Alzheimer’s disease, since RAGE located within cerebral vascular membranes mediates the transport of amyloid beta (Aβ) from the blood to the brain, thus supporting the buildup of cerebral amyloid plaques [20,42,43]. Therefore, serum anti-RAGE antibodies are potential agents to block RAGE-mediated Aβ transport into the CNS. Webster previously reported a vaccine formed by a complex comprising a RAGE peptide along with Aβ42 [44]. Interestingly, RAGE has recently been identified as a relevant target in cancer immunotherapy [39].

The biocompatibility, low cytotoxicity, and stability of PSiP have been widely studied in both in vitro [45,46] and in vivo studies [47,48]. Interestingly, Santos et al. [49] studied the effect of particle size, concentration, and the surface chemistry of PSiP on CaCo-2 cells and macrophages. The report considered the effect of particles’ size in polydisperse groups and concluded that smaller particles showed more cytotoxic effects. The cytotoxicity decreased at the lowest concentrations (0.2 mg/mL). Besides, PSiP stabilized by thermal oxidation demonstrated less cytotoxic effects that those PSiP with thermal hydrocarbon oxidation. In the present work, we reported a weekly administration of 0.4 µg/mL PSiP (with thermal oxidation) to test mice, observing no obvious signs of toxicity. Therefore, it is plausible to argue that RAGEp/PSiP is biocompatible, not cytotoxic, and stable in the biomodel used in the present study.

On the other hand, evidence in the literature suggests that the physical forces involved in the PSiP–peptide or protein interactions prevail in in vivo conditions. It has been reported that morphogenetic protein 7 (BMP7) (28 kDa) was totally released from silicon microparticles with pore size >10 nm in 24–48 h, while the same BMP7 was retained in silicon microparticles with pore size <6 nm after 96 h. The authors discussed that protein size is also important, as proteins should be able to enter into particle cavities. In the present study, the average pore size of PSiP was 60 nm; then, the peptide RAGEp (~4 kDa) could be easily accumulated into the pores, and remain as RAGEp-PSiP for at least 24 h [50]. Another study reports that 50% of the peptide Melanotan II is released from PSiP within a period of 15 days [51]. Furthermore, several reports have discussed that proteins and peptides loaded in porous particles continue to be pharmacologically active in in vivo assays [52,53]. Thus, it is reasonable to suggest that RAGE-PSiP remains as a conjugate for at least 24 h thanks to the favorable molecular interactions between RAGEp and PSiP at the physiologic pH; these interactions also allow the uptake of the particulate complexes by antigen-presenting cells, which enhances the immunogenic activity of RAGEp (see Figure 7).

Therefore, RAGE is a major therapeutic target for inhibiting the pathophysiological consequences of the ligand/RAGE complex. Conventional drugs that act as an antagonist of RAGE ligands have been developed and evaluated in Phase II clinical trials for AD [54] and diabetes nephropathy [55], rendering them as ineffective candidates. Immunotherapies no doubt constitute an alternative to achieve a proper RAGE blockage through epitope-based vaccines that offer high specificity.

## 4. Materials and Methods

### 4.1. Electrochemical Etching Synthesis of PSiP

Silicon wafers Si (100) (WRS Materials, CA, USA) of p+ type doped with boron and resistivity values of 0.01–0.1 Ω∙cm were used in the preparation of porous silicon (PSi). The PSi was prepared by electrochemical etching in a fluorhydric acid (48%, Golden Bell Inc., Anaheim, CA, USA) and ethanol (>98%, Sigma Aldrich, Toluca, Mexico) mixture (HF:EtOH 3:7). A current density of 46 mA/cm^2^ was used to obtain a porosity of about 70%. The resulting films were removed from the silicon substrate by electropolishing with a current density of 180 mA/cm^2^ for 2 s, and the process was repeated three times. Afterwards, the free-standing films were placed in ethanol; the liquid was sonicated using an ultrasonic processor for periods of 1 min. The sonication cycles were repeated 10 times to achieve the desired particle size distribution. After sonication, the PSiP were thermally oxidized at 400 °C with air for 1 h. Then, the PSiP were stored until further characterization and evaluation.

### 4.2. PSiP Characterization

The PSiP were characterized by scanning electron microscopy (SEM, ESEM FEI-QUANTA 200) to analyze the morphology and pores distribution on the surface (ThermoFisher Scientific, Hillsboro, OR, USA). Both the particle and pore sizes of PSiP were obtained from the SEM images using ImageJ software (Version 1.50, National Institutes of Health, Bethesda, MD, USA). The surface charges of PSiP alone and loaded with the synthetic RAGE peptide were determined by measuring the zeta potential (ZP) at different pH values using a Malvern Zetasizer (Malvern Panalytical Ltd., Malvern, UK). Specific functional groups of PSiP were characterized using an infrared spectrophotometer from Agilent Technologies (Cary 600, FT-IR) (Agilent Technologies, Santa Clara, CA, USA).

### 4.3. Adsorption of the Synthetic RAGE Peptide onto the PSiP Surface

A synthetic peptide called RAGEp from the extracellular domain of human RAGE (aa 23–54) [56] was synthesized by GenScript Inc (Piscataway, NJ, USA). (Figure 1). The GenScript Peptide Property Calculator indicates that the isoelectric point (*pI*) of RAGEp is 10.62, and possesses a total charge of +4 with a basic character (http://www.genscript.com). For adsorption experiments, 100 μg of PSiP were mixed with different amounts of RAGEp (25 μg, 40 μg, and 60 μg) and 250 μL of phosphate buffered-saline (PBS) solution (pH = 7.4). All of the suspensions were placed at 6 °C under constant stirring during the experiment. The concentration of soluble RAGEp was determined as follows: at specified times, the suspensions were centrifuged at 4000 rpm for six minutes at 6 °C to withdraw 6 μL aliquots from the supernatant, which were placed on a ultraviolet–visible spectrophotometer (UV-Vis) Tray Cell (λ_max_ = 199 nm) to determine absorbance. Concentrations were calculated by interpolation of the absorption values using a RAGEp calibration curve (λ_max_ = 199 nm, R^2^ = 0.999, data not shown). These aliquots were returned to the original RAGEp/PSiP suspensions after UV-Vis measurements. The experiment ended when three consecutive soluble RAGEp concentrations changed by no more than 3%. All of the solutions were analyzed by triplicate. After reaching equilibrium, the suspensions were centrifuged using the same previous conditions, and the obtained RAGEp/PSiP conjugate pellets were analyzed by FT-IR and compared with both RAGEp and PSiP alone. The percentage of RAGEp adsorbed onto the PSiP surface (%_RAGEp/PSiP_) or loading degree was calculated using the following equation:

$$\;{\%}_{\mathrm{RAGEp}/\mathrm{PSiP}}=\left(m_{0,\mathrm{RAGEp}}-m_{\mathrm{sol},\mathrm{RAGEp}/\mathrm{PSiP}}/m_{0,\;\mathrm{RAGEp}}\right)\;\times 100\%$$

where m_sol,RAGEp/PSiP_ is the amount of soluble RAGEp (μg) at any time.

### 4.4. Immunogenicity Assay

For this study, the experimental procedures in mice were approved by the Institutional Animal Care and Use Committee (Protocol number: CEID-2015-069). Five groups (*n* = 3) of six to eight-week-old, female BALB/c mice were established; they received four weekly subcutaneous immunizations of one of the following treatments: 200 μL PBS, 25 μg of RAGEp, 25 μg/100 μg of RAGEp/PSiP, 100 μg of PSiP, or 25 μg/100 μL RAGEp/CFA/IFA (CFA: complete Freund’s adjuvant; IFA: incomplete Freund’s adjuvant). RAGEp (1 μg μL^−1^) was maintained at −40 °C until dose preparation. The RAGEp/PSiP vaccine was prepared by adding an excess of RAGEp (60 μg) into a PBS solution with 100 μg of PSiP for each dose. The suspension was under constant stirring at 6 °C for 4 h. Afterwards, the suspension was centrifuged at 4000 rpm for six minutes, and the supernatant was removed. The RAGEp/PSiP pellet was resuspended in 200 μL of PBS per dose. All of the vaccines had a dose volume of 200 μL. Mice were bled before the first immunization, and on days 7 and 52 after the first boost, in order to conduct ELISA to determine anti-RAGEp IgG levels.

ELISA was conducted using 90 six-well polystyrene plates coated overnight with RAGEp (0.25 μg/well) at 4 °C. After blocking with 5% fat-free milk for 2 h, the plates were incubated overnight at 4 °C with serial dilutions of mice sera (1:20, 1:40, and 1:80). Anti-IgG horseradish peroxidase-conjugated secondary anti-mouse antibodies (1:2000 dilution, Sigma-Aldrich Inc., Saint Louis, MO, USA.) were applied for 2 h at room temperature and, after washing with PBS-Tween buffer, the signals were detected following incubation with a 2,2′-azino-bis(3-ethylbenzothiazoline-6-sulphonic acid) (ABTS) substrate (Sigma-Aldrich Inc., Saint Louis, MO, USA.) and 0.1 mM H_2_O_2_ (Sigma-Aldrich Inc.) for 15 min. Optical density values were measured at 405 nm using a microplate reader (Thermo Fisher Scientific, Hampton, NH, USA). Significant differences in the antibody levels between groups were assessed using one-way analysis of variance (ANOVA) followed by mean comparisons applying Tukey’s test (*p* < 0.05). Statistical analyses were performed using the Minitab software (Version 1.5.0, Minitab Inc., State College, PA, USA).

## 5. Conclusions

PSiP enhances the efficacy of immunization against RAGE, leading to long-lived humoral responses. This constitutes the first evidence of the adjuvanticity of PSiP upon the direct immunization of test animals, and opens the path for the development of vaccines based on PSiP as an adjuvant/carrier.

## Figures and Tables

**Table 1:** IR signals of RAGEp-PSiP conjugate and possible intermolecular forces involved in the physical interaction.

Wavenumber (cm^−1^)	IR Signal Presenting a Change in Intensity with Respect to PSiP IR Signals	Possible Intermolecular Force
3370	O–H stretching signal of Si–O–H bond [33]	Electrostatic
2150	OSiH_3_ stretching band [26,27]	Ion–dipole
1066	Si–O–Si stretching signal. Broad band with a shoulder at 1215 cm^−1^ [26,27].	Ion–dipole
767	O_n_Si–H_x_ deformational vibration [23,24].	Electrostatic/ion–dipole
